# Arthroscopic rotator cuff repair in patients over 70 years of age: a systematic review

**DOI:** 10.1186/s10195-021-00565-z

**Published:** 2021-02-18

**Authors:** Chiara Fossati, Carlo Stoppani, Alessandra Menon, Luca Pierannunzii, Riccardo Compagnoni, Pietro S. Randelli

**Affiliations:** 1 Laboratory of Applied Biomechanics, Department of Biomedical Sciences for Health, Università degli Studi di Milano, Via Mangiagalli 31, 20133 Milan, Italy; 2 U.O.C. 1a Clinica Ortopedica, ASST Centro Specialistico Ortopedico Traumatologico Gaetano Pini-CTO, Piazza Cardinal Ferrari 1, 20122 Milan, Italy; 3 Department of Biomedical Sciences for Health, Research Center for Adult and Pediatric Rheumatic Diseases (RECAP-RD), Università degli Studi di Milano, Via Mangiagalli 31, 20133 Milan, Italy

**Keywords:** Rotator cuff tear, Arthroscopic rotator cuff repair, Rotator cuff repair in elderly, Patients over 70 years of age, Shoulder arthroscopy

## Abstract

**Background:**

Failure of conservative treatment in patients over 70 years of age with a rotator cuff tear makes surgery a possible option, considering the increase in life expectancy and the high functional demands of elderly patients. The purpose of this systematic review of the literature was to evaluate the subjective and objective outcomes after arthroscopic rotator cuff repair in patients over 70 years of age.

**Methods:**

A systematic review was performed to identify all the studies reporting subjective and objective outcomes in patients aged 70 years or older undergoing arthroscopic rotator cuff repair. Constant Murley Score (CMS), visual analog scale (VAS), American Shoulder and Elbow Surgeons Score (ASES), and Simple Shoulder Test (SST) were used to detect any clinical improvement after surgery. Retear and satisfaction were also analyzed.

**Results:**

Out of 941 studies identified, only 6 papers have been included in the review. All studies reported improvements in postoperative functional outcome scores that exceed the minimal clinically relevant difference. The mean retear rate amounts to 21.9%, which is in line with the failure rate of rotator cuff repair in general population. Moreover, postoperative satisfaction is very high (95%).

**Conclusion:**

This systematic review suggests that arthroscopic rotator cuff repair in patients over 70 years of age could be a valid treatment option after failure of conservative approach.

**Level of evidence:** 4

*Trial registration* The study was registered on PROSPERO (registration ID: CRD42018088613)

## Introduction

Rotator cuff (RC) tears are a common cause of pain and disability of the shoulder in adult population, with an incidence that increases with age. In fact, although a genetic susceptibility seems to be present [1], the RC lesions basically result as part of a degenerative process of aging [2, 3].

Radiologic studies revealed that the prevalence of asymptomatic full-thickness RC tears is 28% in patients ≥ 60 years and 50% in patients ≥ 70 years [4, 5]. Moreover, 50% of these asymptomatic tears seem to become symptomatic at a mean of 2.8 years after diagnosis [6].

In older patients, primary conservative treatment for symptomatic RC tears is a reasonable option, as shown by the good clinical results for this population [7, 8].

Moreover, surgical treatment in elderly patients could be insidious since advanced age has been identified as a negative prognostic factor for RC healing with a retear rate of 32% in patients older than 70 years [9, 10]. Low healing response in older patients may be due to several reasons that potentially increase the difficulty of repair: larger lesions, fatty degeneration, and poor quality of tendon [2, 9, 11]. Furthermore, age-related comorbidities may frequently compromise surgical treatment. In fact, older patients are often affected by osteoporosis, which can be responsible for anchor pullout or tuberosity fracture [4], and metabolic syndrome, which is reported to reduce tendon healing [12, 13].

However, failure of conservative therapy in this population makes surgical treatment of RC tears a valid option of treatment, considering the increase in life expectancy and the high functional demands of many patients > 70 years [14]. Recently, several authors have started to study the results of RC repair in elderly patients.

The purpose of this systematic review of the literature was to evaluate the subjective and objective outcomes after arthroscopic RC repair in patients over 70 years of age.

## Materials and methods

**Fig. 1:**
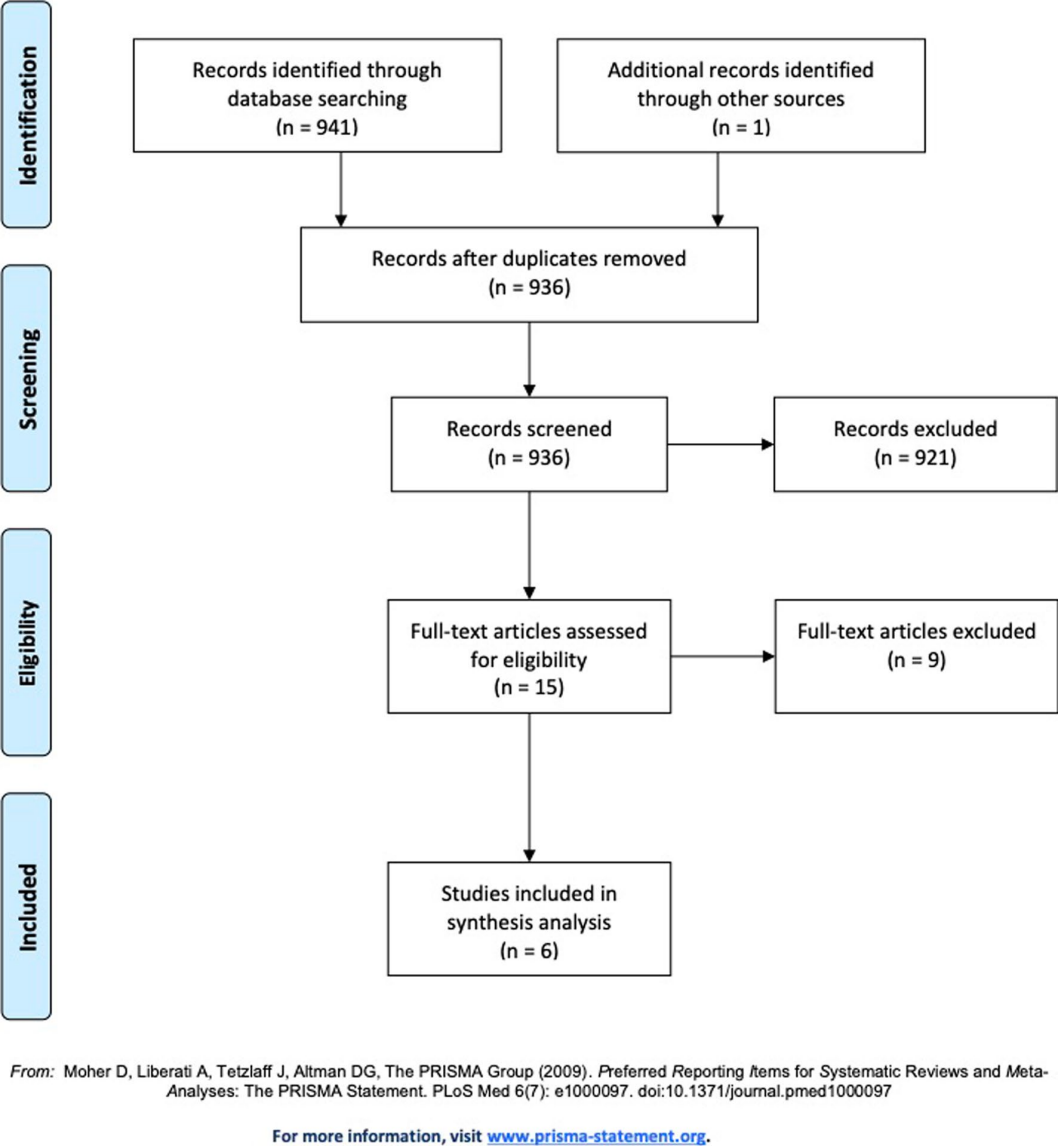
Flowchart of literature review

A systematic review was performed to identify all studies reporting subjective and objective outcomes in patients aged 70 years or older undergoing arthroscopic RC repair. The Preferred Reporting Items for Systematic Reviews and Meta-Analyses (PRISMA) guidelines were followed to perform this systematic review of the literature and to present the results [15]. A protocol was written stating the purpose of the review, and the search strategy and was registered on PROSPERO on February 27, 2018 (registration ID: CRD42018088613). A flow diagram according to PRISMA guidelines summarizes our selection protocol (Fig. 1).

### Searches

An electronic search of the literature was performed in the MEDLINE database via PubMed and Embase database from the databases’ inception up to 26 November 2020, using the following search string for title and abstract: (((Rotator Cuff OR Rotator Cuff Injuries OR Rotator Cuff Tear Arthropathy) AND (repair) AND (Arthroscopy OR arthroscopic)) NOT ((platelet-rich plasma) OR (prp)) OR ((Rotator Cuff OR Rotator Cuff Injuries OR Rotator Cuff Tear Arthropathy) AND (repair) AND (Arthroscopy OR arthroscopic)) NOT ((miniopen OR mini + open OR open))) LIMITS (aged OR aged,70 and more). MeSH terms were used for “Rotator Cuff”, “Rotator Cuff Injuries”, “Rotator Cuff Tear Arthropathy”, and “Arthroscopy”.

The search was restricted to English-language literature; meta-analyses, systematic reviews, and narrative reviews were excluded.

### Study inclusion and exclusion criteria

According to the methodology recommended by Harris et al. [16], after deletion of duplicates, title and abstract of all identified studies were independently examined by two reviewers, who applied the study eligibility criteria.

In particular, the inclusion criteria were English language and level of evidence 1 to 5 that evaluated the subjective and objective outcomes after arthroscopic RC repair in patients aged 70 years or over.

Exclusion criteria were not meeting inclusion criteria, reviews, narrative reviews, systematic reviews, meta-analyses, and clinical studies that evaluate open or mini-open RC repair in patients aged 70 years or older, or arthroscopic RC repair with the use of platelet-rich plasma augmentation. In addition, epidemiological, radiological, animal, and cadaveric studies were excluded.

In case of disagreement between reviewers, consensus was sought through discussion and, in case of persistent disagreement, a third reviewer was consulted and the study was included until full-text review could be performed. Finally, eligible articles underwent full-text review for a more detailed evaluation. Both reviewers also manually cross-referenced to ensure that all potential studies were included. Reviewers were not blinded to the authors or affiliated institutions of the retrieved studies. The final list of included studies was agreed to by consensus.

### Study quality assessment

Two reviewers (C.F. and C.S.) independently assessed methodological quality of the included studies according to the Methodological Index for Nonrandomized Studies (MINORS) checklist [17]. On the basis of this tool, 8 items for noncomparative studies and 12 items for comparative studies have been evaluated with a score that varies from 0 to 2 (0, not reported; 1, reported but inadequate; 2, reported and adequate). Therefore, the maximum global score was 16 for a noncomparative study and 24 for a comparative study.

The level of evidence of each article was assessed using the 2003 *Journal of Bone and Joint Surgery* definitions for orthopedic publications [18].

### Data extraction strategy

Two reviewers independently (C.F. and C.S.) extracted study data using a standardized data extraction form that was predefined according to the protocol. Discordance was resolved by both reviewers rechecking their extracted data until data sheets corresponded. If no consensus could be reached, a third reviewer (AM) was consulted. When presented, for each study, information regarding the characteristics of the studies (author, year and journal of publication, study design and level of evidence, number of patients and shoulders), the characteristics of the participants of the studies (age, dominant shoulder or not, follow-up duration, preoperative validated outcome measures), and the clinical outcome of the treatment (postoperative outcome measures, failure rates and evidence of tendon healing, clinical results of the final follow-up) was extracted. Where possible, the compiled data from individual studies with the same outcome measures were pooled together. Demographic data were compiled to assess weighted mean ages across groups.

## Results

As shown in Fig. 1, our search identified 941 studies based on the described searching strategy protocol.

After a careful screening of title and abstract, 15 articles underwent full-text review for a more detailed evaluation. The strict eligibility criteria applied in this review finally reduced the number of articles to six studies [10, 19–23].

The characteristics of the included studies are presented in Table 1.

**Table 1:** Studies included in the review

Authors	Year	Study design	Level of evidence	No. of patients (shoulders)	Sex, no. (male/female)	Age (years), mean ± SD	Outcomes	Size of cuff tear	Final follow-up (months), mean ± SD	MINORS quality score
Bhatia et al. (*Am. J. Sports Med.*)	2015	Case series	IV	44 (43 reviewed at final follow-up)	33/11	73 ± 3.2 (70–82)	Clinical outcomes: ASES, SANE, QuickDASH; SF-12 PCS; Satisfaction radiological outcomes: Not described	Large/massive tears: 69%	43.2 (24–94.8)	11/16
Flurin et al. (*Orthop. Traumatol. Surg. Res.*)	2013	Multicenter prospective study	II	145 (135 reviewed at final follow-up)	66/79	73.9 ± 3.4 (70–82)	Clinical outcomes: VAS, ASES, SST, Constant Murley Score. Radiological outcomes: Ultrasound	No large/massive tears	12 (min follow-up)	10/16
Miyazaki et al. (*Rev. Bras. Ortop.*)	2015	Retrospective study	III	163 (168)	63/100	70.8 (65–83)	Clinical outcomes: UCLA, Satisfaction. Radiological outcomes: Not described	Large tears: 44.2% (age 70–74) 50% (age > 75)	50.6 (12–144)	7/16
Moraiti et al. (*Arthroscopy*)	2015	Therapeutic case series	IV	40	13/27	73 (70–83)	Clinical outcomes: Constant Murley Score, VAS, Satisfaction. Radiological outcomes: Ultrasound	–	13.8 (12–32)	19/24
Robinson et al. (*Bone Joint J.*)	2013	Retrospective study	III	68 (62 reviewed at final follow-up)	33/35	77 (70–86)	Clinical outcomes: Constant Murley Score, Radiological outcomes: Ultrasound	Large/massive tears: 68.1%	Median 14 (1–50)	9/16
Verma et al. (*Arthroscopy*)	2010	Therapeutic case series	IV	44 (39 reviewed at final follow-up)	18/21	75.3 ± 4.2 (70.1–89.8)	Clinical outcomes: VAS, ASES, SST, Constant Murley Score, SANE, Satisfaction. Radiological outcomes: Not described	Large/massive tears: 17.9%	36.1 ± 9.9 (24.3–59.4)	9/16

Year of publication, study design, level of evidence, and patient information are described

AP, anteroposterior; ASES: American Shoulder and Elbow Surgeons Score; min, minimum; MINORS, Methodological Index for Nonrandomized Studies; MRI, magnetic resonance imaging; No., number; QuickDASH, Disability of the Arm, Shoulder and Hand; SANE, Single Assessment Numeric Evaluation; SD, standard deviation; SF-12 PCS, Short Form-12; SST, Simple Shoulder Test; UCLA, The University of California at Los Angeles Shoulder Score; VAS, visual analog scale

**Fig. 2:**
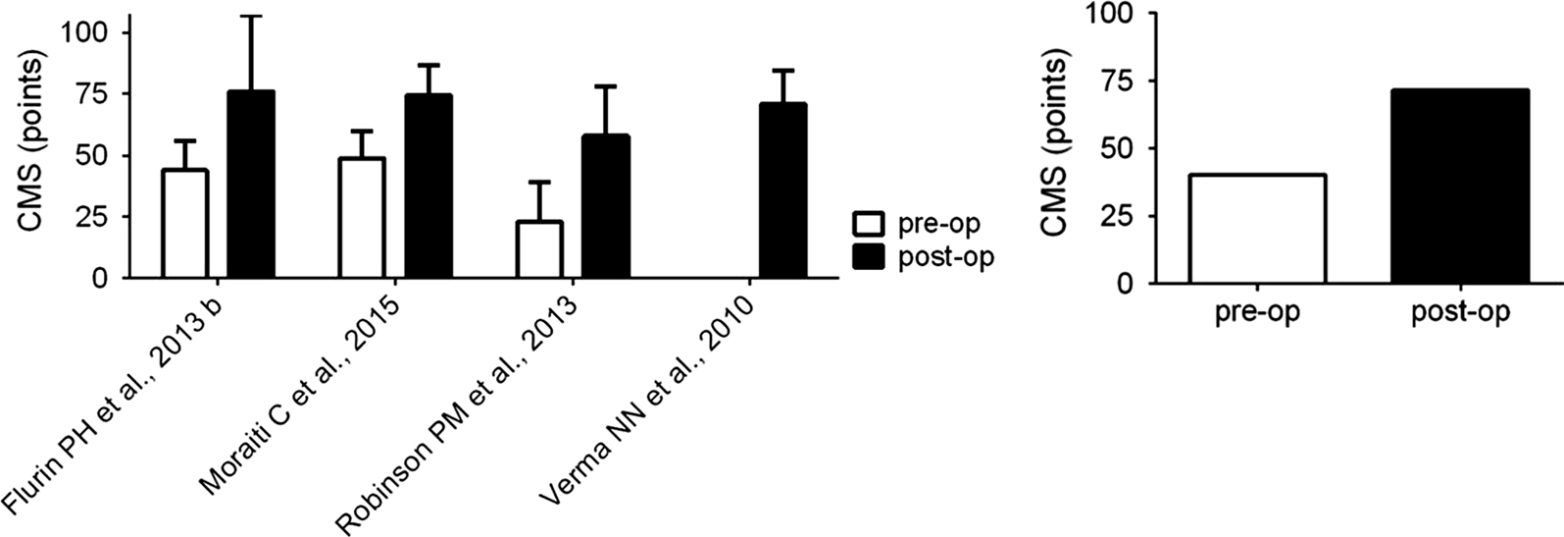
Comparison of CMS in pre- and postoperative populations. Left: mean CMS values reported in the analyzed studies; Right: weighted average value of CMS. CMS, Constant Murley Score; pre-op, preoperative; post-op, postoperative

The mean follow-up was 28 months with a wide range (12–50 months). As shown in “Materials and methods” section, different scores were evaluated since there is not a common tool for the clinical evaluation: Constant Murley Score (CMS), visual analog scale (VAS), American Shoulder and Elbow Surgeons Score (ASES score), and Simple Shoulder Test (SST). The CMS was assessed at the postoperative follow-up in four studies (265 shoulders): the mean value was 71.5 points (range 58.0–76.0 points). Of four studies analyzed, only three reported the preoperative CMS (236 shoulders) with a mean preoperative value of 40.3 points (range 23.0–48.8 points). The CMS improved after surgery with a mean value of improvement of 31.2 points (Fig. 2). Considering the minimal clinically important difference for CMS (10.4 points) [24], the overall analysis of the reported data showed a relevant increase in the mean CMS after surgery.

**Fig. 3:**
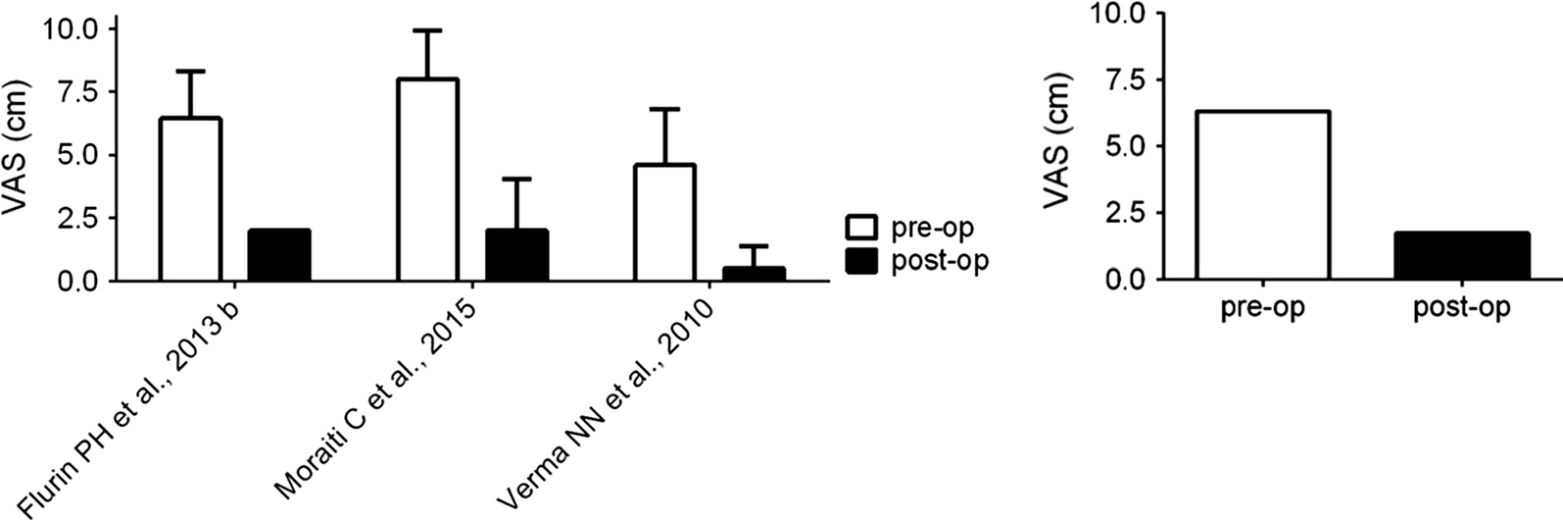
Comparison of VAS in pre- and postoperative populations. Left: mean VAS values reported in the analyzed studies; Right: weighted average value of VAS. VAS, visual analog scale; pre-op, preoperative; post-op, postoperative

The VAS score was evaluated in three studies (Fig. 3). The overall analysis of postoperative VAS score revealed a significant difference as compared with the preoperative values: in particular, this score decreased after surgery from 6.3 cm (range 4.6–8.0 cm) to 1.7 cm (range 0.5–2.0 cm). The difference of 4.6 cm is almost three times the minimal clinically important difference for VAS (1.4 cm) [25.]

**Fig. 4:**
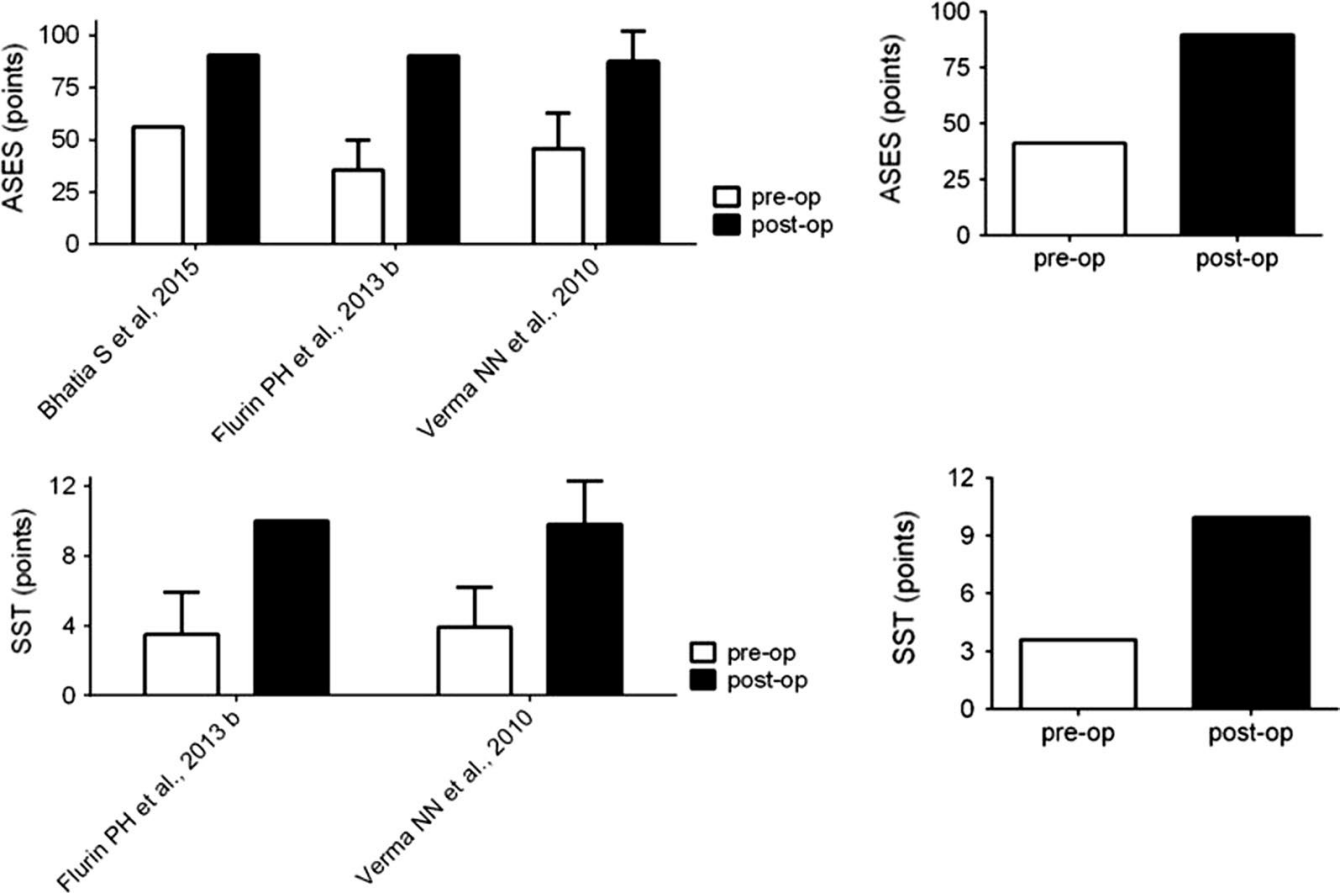
Comparison of ASES and SST in pre- and postoperative populations. Left: mean ASES and SST values reported in the analyzed studies; Right: weighted average value of ASES and SST. ASES, American Shoulder and Elbow Surgeons Score; SST, Simple Shoulder Test; pre-op, preoperative; post-op, postoperative

Similar improvements were registered in ASES and SST score as shown in Fig. 4.

Retears of the RC were evaluated with ultrasound in three studies (237 patients). According to these studies, 21.9% (range 17.5–32%) of the patients presented a new lesion at follow-up.

In one study [20], patients with full-thickness reruptures had a significantly lower Constant Score (77 versus 70; *p* = 0.015) and ASES (91 versus 82; *p* = 0.02). Also, Robinson et al. [10] showed a greater improvement of the postoperative Constant Score in patients with intact rotator cuff at follow-up (43 versus 14 points), but there is no information about significance level.

Satisfaction, reported in three studies [21–23], was, on average, very high: the mean value of satisfied patients at final follow-up was 95% (range 93–97%).

## Discussion

The principal finding of the present study is that arthroscopic RC repair in elderly population over 70 years of age provides good improvement in shoulder function with a very high rate of patient satisfaction (95%). On average, more than 450 patients were evaluated in this review, and the satisfactory results of the study justify the surgical approach in elderly population.

In literature, the effect of advanced age on RC healing is still debated, and 69 years of age is identified as a conventional cutoff value for successful healing after arthroscopic repair in small- to medium-sized RC tears [26]. Indeed, the retear rate, which increases minimally until 65 years of age, starts to rise substantially over the age of 70 years, probably because older patients frequently present large and complete tears upon surgery [2]. In fact, Miyazaki et al. confirmed that extensive lesions were greater in older population (37.5% among patients up to 69 years of age and 50% among those aged over 75 years). [21].

Nevertheless, despite the assumed poorer tendon healing capacity of older patients, the studies included in this systematic review showed improvement in all clinical and functional scores.

RC healing was assessed in only four studies with MRI or ultrasound [10, 20, 21, 23]. We found a mean retear rate of 18.6%, which is similar to the data reported by Diebold in his study of 1600 arthroscopic RC repairs. They reported an overall failure tendon healing in 13% of patients, with a retear rate of 25% in those aged 70 to 79 years [27].

Good and comparable outcomes are found even when RC tears in patients over 70 years are treated with an open technique [28–30]: De Carvalho et al. [28] show how the average postoperative Constant Score was 80.1 and the mean SST was 9.8. Nevertheless, arthroscopic RC repair is minimally invasive, with a lower risk of deltoid damage, allows treatment of associated lesions, and, potentially, results in faster recovery [31, 32].

Several limitations affect this study: firstly, the different clinical outcome scores used in the selected articles reduced the sample for quantitative analysis. In addition, there is no information about relevant clinical factors such as grade of fatty infiltration, treatment history, surgical technique (single-row, double-row, side-to-side sutures), or accessory surgical treatment (biceps tenotomy or tenodesis, acromioplasty, or distal clavicle resection). Moreover, our review is exposed to some bias because of the low level of the methodological quality of the studies included (no RCT) and the unavailability of raw data.

In conclusion, arthroscopic rotator cuff repair in patients over 70 years of age showed good clinical results and high satisfaction rate and can thus be considered a valid option of treatment after failure of conservative approach. The result of this review should encourage future randomized controlled studies to focus on this population to support surgical treatment also in elderly patients.

## Data Availability

All data generated or analyzed during this study are included in this published article.

## Abbreviations

CMS: Constant Murley Score
VAS: Visual analog scale
ASES: American Shoulder and Elbow Surgeons Score
SST: Simple Shoulder Test
RC: Rotator cuff
